# Supplementary material for: Predicting lapses of attention with sleep-like slow waves
Source: Nat Commun. 2021 Jun 29;12:3657. doi: 10.1038/s41467-021-23890-7 (PMC8241869; doi:10.1038/s41467-021-23890-7)
Supplement: Supplementary file 1 — Supplementary Information [file 41467_2021_23890_MOESM1_ESM.pdf]

Supplementary Information:

Predicting lapses of attention with sleep-like slow waves.

Thomas Andrillon[1,2]*, Angus Burns[1], Teigane MacKay[1], Jennifer Windt[3] & Naotsugu Tsuchiya[1,4,5]

Affiliations:

1. School of Psychological Sciences, Turner Institute for Brain and Mental Health, Monash University, Melbourne 3168, Victoria, Australia.
2. Institut du Cerveau - Paris Brain Institute - ICM, Sorbonne Université, Inserm, CNRS, Paris 75013, France
3. Philosophy Department, Monash University, Melbourne 3168, Victoria, Australia.
4. Center for Information and Neural Networks (CiNet), National Institute of Information and Communications Technology (NICT), Suita, Osaka 565-0871, Japan
5. Advanced Telecommunications Research Computational Neuroscience Laboratories, 2-2-2 Hikaridai, Seika-cho, Soraku-gun, Kyoto 619-0288, Japan.

Supplementary Methods

Supplementary Table 1 to 3

Supplementary Figures 1 to 12

Supplementary Methods

Participants. Prior to their participation in the protocol, all 26 participants but one filled in online surveys on Qualtrics (N=25). They reported normal levels of sleepiness (Epworth Sleepiness Scale: 14.6 ± 4.7; mean ± standard-deviation) and mind wandering (Mind Wandering Questionnaire[1]: 3.6 ± 0.91) in their everyday lives. Participants were not instructed to follow a particular schedule before the experiment (in particular, our protocol did not involve a sleep restriction procedure). The timing of the experiment was determined by participants' availability and included both morning and afternoon sessions.

Experimental Design and Stimuli. Face stimuli were divided in two parts vertically (half-left and half-right faces) which were flickered on the screen at different frequencies (12 and 15 Hz, counterbalanced across participants). Similarly, the digits were inserted in a Kanizsa illusory square (Fig. 1a) whose right and left parts also flickered at different frequencies (12 and 15 Hz). This flickering was introduced to elicit Steady State Visual Evoked Potentials (SSVEPs) in the EEG signal. The detailed analysis of this aspect of our dataset will be reported elsewhere. As the flickering occurred at a high rate, participants did not report a negative effect on their ability to perform the SART.

Experience Sampling. Following task interruptions (probes), participants were asked to answer a series of 8 questions in the following fixed order: (1) "Were you looking at the screen?" (response: yes / no); (2) "Where was your attention focus?" (response: on-task / off-task / blank / don't remember); (3) "What distracted your attention from the task?" (response: Something in the room / personal / about the task); (4) "How aware were you of your focus?" (response: from 1, I was fully aware, to 4, I was not aware at all); (5) "Was your state of mind intentional?" (response: from 1, entirely intentional, to 4, entirely unintentional); (6) "How engaging were your thoughts?" (response: from 1, not engaging, to 4, very engaging); (7) "How

well do you think you have been performing?" (response: from 1, not good, to 4, very good); (8) "How alert have you been?" (response: extremely alert / alert / sleepy / extremely sleepy). Question 3 was displayed only if participants answered off-task in question 1. In this report, we focus only on questions (2) and (8).

Physiological Recordings and Preprocessing. The raw pupil size was corrected for the occurrence of blinks as in[2]. The timings of blinks were obtained through the EyeLink acquisition software. For each of these blinks, the pupil size was corrected by linearly interpolating the average signal preceding the blink onset ([-0.2, -0.1]s) and following the blink offset ([0.1, 0.2]s). The corrected signal was then low-pass filtered below 6Hz (two-pass Butterworth filter at the 4th order). Finally, for blinks longer than 2s, data points between -0.1s prior to blink onset and 0.1s after blink onset were considered missing.

Detection of Slow Waves. In sleep, according to established guidelines[3], only waves with peak-to-peak amplitude exceeding 75μV are defined as slow waves. In wakefulness, similar slow waves can be observed but with smaller amplitudes. Accordingly, previous studies relied on a relative rather than absolute threshold[4–6]. Here, we defined slow waves as the waves with absolute peak-to-peak amplitude within the top 10% of all the waves detected for a given EEG electrode and a given participant. On average, the detection threshold was 30 μV (average across N=26 participants and across all electrodes).

Figure 3a shows the average waveform of the slow waves detected on electrode Cz as well as the average waveform of waves detected during sleep recording in another published dataset (N=15 participants)[7]. To compute the average waveform of sleep slow waves, we applied the same algorithm to epochs of 20s scored as NREM2 and NREM3. Only slow waves with peak-to-peak amplitude over 75μV were considered.

We also compared the average waveform of slow waves with task-related Event Related Potentials (ERPs). To do so, we averaged the EEG signal time-locked to stimuli or response onsets at the electrode level. Supplementary Figure 11a-c shows the average ERP across participants for electrode Cz as well as the scalp topographies of the voltage observed across the scalp at the time of the peak for electrode Cz. Finally, we also compared single-trial voltage values between the average waveforms for slow waves and stimulus-locked or response-locked ERPs (Supplementary Figure 11d-f, data shown for one participant). Overall, these analyses showed a clear difference between the amplitude, waveform and topographies of slow waves compared to task-related ERPs.

Drift Diffusion Modelling. The Drift Diffusion Model (DDM) assumes that a decision variable noisily accumulates evidence from a starting point (z) with drift rate (v) towards one of two boundaries that represent choice alternatives (i.e. Go or NoGo; see Supplementary Figure 7). The decision threshold (a) is the distance between the two boundaries and represents the amount of evidence that must be accumulated before a decision is made. Once the decision variable crosses a decision boundary, a response is made. Five parameters were fitted using a DDM approach: the participants' initial bias for one of the two responses (decision bias, z), drift rates for Go and NoGo responses (v_{Go} and v_{NoGo}), the decision threshold (a), the non-decision time parameter (t). The last parameter, t, captures extra-decisional components, including stimulus encoding, response preparation and execution. We also extracted the drift rate bias (v_{Bias}) as the difference between the absolute values of v_{Go} (positive) and v_{NoGo} (negative). We performed a model selection based on the Deviance Information Criteria (DIC), which assess goodness of fit while accounting for model complexity in hierarchical models[8]. With posterior predictive checks, we confirmed that our DDM was able to generate simulated behavioural data that are similar to the recorded data (Supplementary Figure 8; based on the

Fz model). For this check, we simulated 100 datasets based on the posterior distributions of HDDM parameters.

Statistics. A cluster-permutation approach (derived from [9]) was applied to identify significant clusters in topographical maps. Candidate clusters were defined as neighbouring electrodes with a p-value below a threshold (called cluster alpha) of 0.025. For each candidate cluster, we computed the sum of the t-values for all the electrodes belonging to the cluster (which we will refer to as the cluster statistics). We then created permuted datasets by permuting the labels of the predictor within each subject, each task and each electrode (N=1,000 permutations). For each of these permuted datasets, we also identified the candidate cluster with maximal absolute cluster statistics. The cluster statistics from permutations formed a null distribution, against which we compared the cluster statistics from the real dataset. Clusters (real and permuted) with positive and negative cluster statistics were compared separately. A Monte-Carlo p-value was derived from this comparison ($p_{cluster}<0.05$ means that a negative cluster has a cluster statistics below the 5th percentile of the permuted distribution and that a positive cluster has a cluster statistics above the 95th percentile of the permuted distribution). In cases where several cluster-permutations were performed in the same analysis (Fig. 5 and 6), we corrected the Monte-Carlo p-values of the real clusters with the Bonferroni method.

Supplementary Table 1. Summary of Linear Mixed-Effects Models

<table>
<tr><th>Figure</th><th>Predicted Variable X</th><th>Level</th><th>Predictor Of Interest</th><th>Model 0</th><th>Model 1</th></tr>
<tr><td>2a-c</td><td>False Alarms, Misses, Reaction Times</td><td>Probe</td><td>Mental State (MS)</td><td>X ~ 1 + Task + (1 | Subject)</td><td>X ~ 1 + Task + MS + (1 | Subject)</td></tr>
<tr><td>2d-e</td><td>Vigilance Scores, Pupil Size</td><td>Probe</td><td>Mental State (MS)</td><td>X ~ 1 + Task + (1 | Subject)</td><td>X ~ 1 + Task + MS + (1 | Subject)</td></tr>
<tr><td>N/A</td><td>Pupil Size</td><td>Probe</td><td>Vigilance Ratings (Vig)</td><td>X ~ 1 + Task + (1 | Subject)</td><td>X ~ 1 + Task + Vig + (1 | Subject)</td></tr>
<tr><td>N/A</td><td>Vigilance Scores</td><td>Probe</td><td>Slow Wave (SW) Properties (density, amplitude, upward slope and downward slope; average across electrodes)</td><td>X ~ 1 + Task + (1 | Subject)</td><td>X ~ 1 + Task + SW + (1 | Subject)</td></tr>
<tr><td>N/A</td><td>Pupil Size</td><td>Probe</td><td>SW Properties (density, amplitude, upward slope and downward slope; average across electrodes)</td><td>X ~ 1 + Task + (1 | Subject)</td><td>X ~ 1 + Task + SW Properties + (1 | Subject)</td></tr>
<tr><td>4*</td><td>Mental State (binary contrast: MW vs ON; MB vs ON and MB vs MW).</td><td>Probe</td><td>SW Properties (density, amplitude, downward slope and upward slope per electrode)</td><td>X ~ 1 + Task + (1 | Subject)</td><td>X ~ 1 + Task + SW Properties + (1 | Subject)</td></tr>
<tr><td>5*</td><td>False Alarms, Misses, Reaction Times</td><td>Trial</td><td>SW (presence or absence)</td><td>X ~ 1 + Task + (1 | Subject)</td><td>X ~ 1 + Task + SW + (1 | Subject)</td></tr>
<tr><td>6*</td><td>a, t, z, v_{Go}, v_{NoGo}, v_{Bias}</td><td>Subject</td><td>SW (presence or absence)</td><td>X ~ 1 + Task + (1 | Subject)</td><td>X ~ 1 + Task + SW + (1 | Subject)</td></tr>
</table>

*: Analyses corrected for multiple comparison (see Methods and Supplementary Methods).

Supplementary Table 2. Effect of normalisation on behavioural analyses

		Normalised	Before Normalisation
Misses (%) in Go trials	State	**$\chi^2(2)$=33.4 (***, p=5.6x10^{-8})**	**$\chi^2(2)$=36.0 (***, p=1.5x10^{-8})**
	MW vs ON	**-0.0096 [-0.015, -0.0041]**	**-0.011 [-0.016, -0.0052]**
	MB vs ON	**-0.023 [-0.032, -0.015]**	**-0.023 [-0.032, -0.015]**
	MB vs MW	**-0.014 [-0.022, -0.0055]**	**-0.013 [-0.021, -0.0047]**
False alarms (%) in NoGo trials	State	**$\chi^2(2)$=88.5 (***, p<2x10^{-16})**	**$\chi^2(2)$=115.9 (***, p<2x10^{-16})**
	MW vs ON	**-0.24 [-0.29, -0.19]**	**-0.20 [-0.24, -0.17]**
	MB vs ON	**-0.20 [-0.26, -0.19]**	**-0.17 [-0.23, -0.12]**
	MB vs MW	0.064 [-0.014, 0.14]	0.028 [-0.028, 0.084]
RT (s) in Go trials	State	**$\chi^2(2)$=33.4 (***, p=2.6x10^{-5})**	**$\chi^2(2)$=16.9 (**, p=2.2x10^{-4})**
	MW vs ON	-0.0005 [-0.013, 0.011]	-0.0025 [-0.0096, 0.0045]
	MB vs ON	**0.039 [0.022, 0.057]**	**0.019 [0.0088, 0.030]**
	MB vs MW	**0.040 [0.022, 0.058]**	**0.022 [0.011, 0.032]**

The analyses of the effect of mental states (model comparisons and post-hoc contrasts) were consistent even after normalizing Misses, False alarms and Reactions Times (RT) for each participant (N=26) by the average obtained on ON probes (Normalised column). The State row shows the result of the likelihood ratio test (chi-squared value). Stars denote the corrected significance level (Bonferroni correction for 6 likelihood ratio tests: ***: p<0.001, **: p<0.01, *: p<0.05). The estimates of the post-hoc contrasts (MW vs ON, MB vs ON, MB vs MW) obtained from the mixed effect models are also reported, with the corresponding 95% confidence intervals. Bold fonts signal the significant results (p<0.05 for model comparisons, or confidence intervals excluding 0 for the post-hoc contrasts). ON: task-focused; MW: mind wandering; MB: mind blanking.

Supplementary Table 3. Effect of different pre-probe window sizes on behavioural analyses

		20s before Probes	10s before Probes
Misses (%) in Go trials	State	**$\chi^2(2)=36.0$ (***, $p=1.5\times10^{-8}$)**	**$\chi^2(2)=31.3$ (***, $p=1.6\times10^{-7}$)**
	MW vs ON	**-0.011 [-0.016, -0.0052]**	**-0.012 [-0.019, -0.0037]**
	MB vs ON	**-0.023 [-0.032, -0.015]**	**-0.033 [-0.045, -0.021]**
	MB vs MW	**-0.013 [-0.021, -0.0047]**	**-0.021 [-0.033, -0.0096]**
False alarms (%) in NoGo trials	State	**$\chi^2(2)=115.9$ (***, $p<2\times10^{-16}$)**	**$\chi^2(2)=55.97$ (***, $p=7.0\times10^{-13}$)**
	MW vs ON	**-0.20 [-0.24, -0.17]**	**-0.20 [-0.25, -0.15]**
	MB vs ON	**-0.17 [-0.23, -0.12]**	**-0.14 [-0.22, -0.062]**
	MB vs MW	0.028 [-0.028, 0.084]	0.061 [-0.018, 0.14]
RT (s) in Go trials	State	**$\chi^2(2)=16.9$ (**, $p=2.2\times10^{-4}$)**	**$\chi^2(2)=8.42$** (ns, $p=0.015$)
	MW vs ON	-0.0025 [-0.0096, 0.0045]	0.0026 [-0.0066, 0.012]
	MB vs ON	**0.019 [0.0088, 0.030]**	**0.020 [0.0064, 0.034]**
	MB vs MW	**0.022 [0.011, 0.032]**	**0.018 [0.0038, 0.031]**

The analyses of the effect of mental states (model comparisons and post-hoc contrasts) were replicated using a shorter window of 10s (right column) instead of 20s before probe onsets (left column). Likelihood Ratio Tests are reported for the effect of State on the correctness for Go and NoGo trials as well as the Reaction Times (RT) for Go Trials. Stars denote the corrected significance level (Bonferroni correction for 6 comparisons: ***: p<0.001, **: p<0.01, *: p<0.05). The estimates for the post-hoc contrasts and the corresponding 95% confidence intervals are also shown. Bold fonts signal the significant results (p<0.05 for model comparisons, or confidence intervals excluding 0 for the post-hoc contrasts). ON: task-focused; MW: mind wandering; MB: mind blanking.

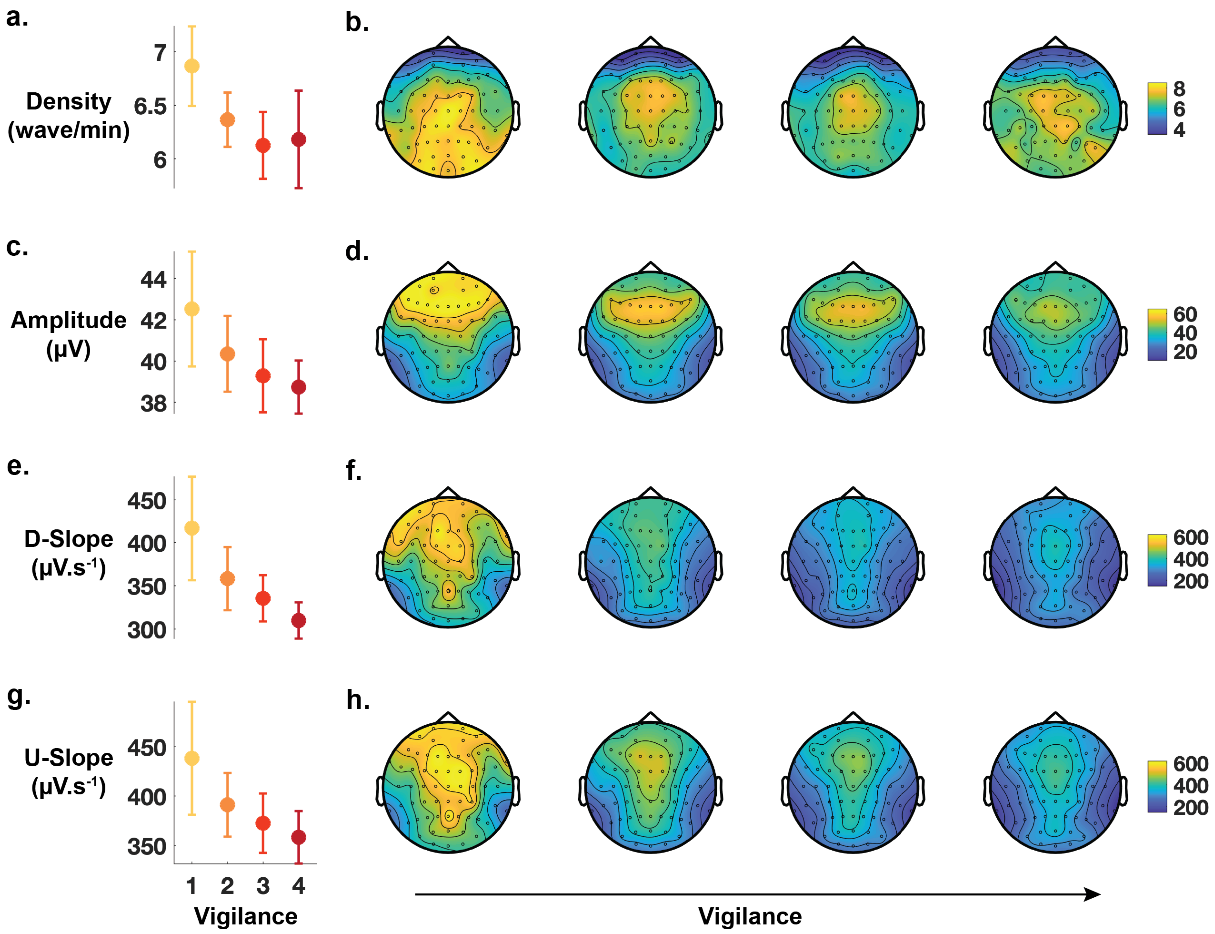

Supplementary Figure 1. Sleepiness is associated with more, larger and steeper slow waves

Slow waves detected within 20s of probes' onset were separated according to participants' vigilance ratings (from 1 (extremely sleepy) to 4 (extremely alert)) provided after each probe. The density (a), amplitude (c), downward slope (D-Slope, e) and upward slope (U-Slope, f), averaged across participants (N=26) and electrodes, are shown with error-bars showing the standard-error-of-the-mean across participants. Topographies of the average density (b), amplitude (d), downward slope (f) and upward slope (h) are also shown for the different vigilance levels.

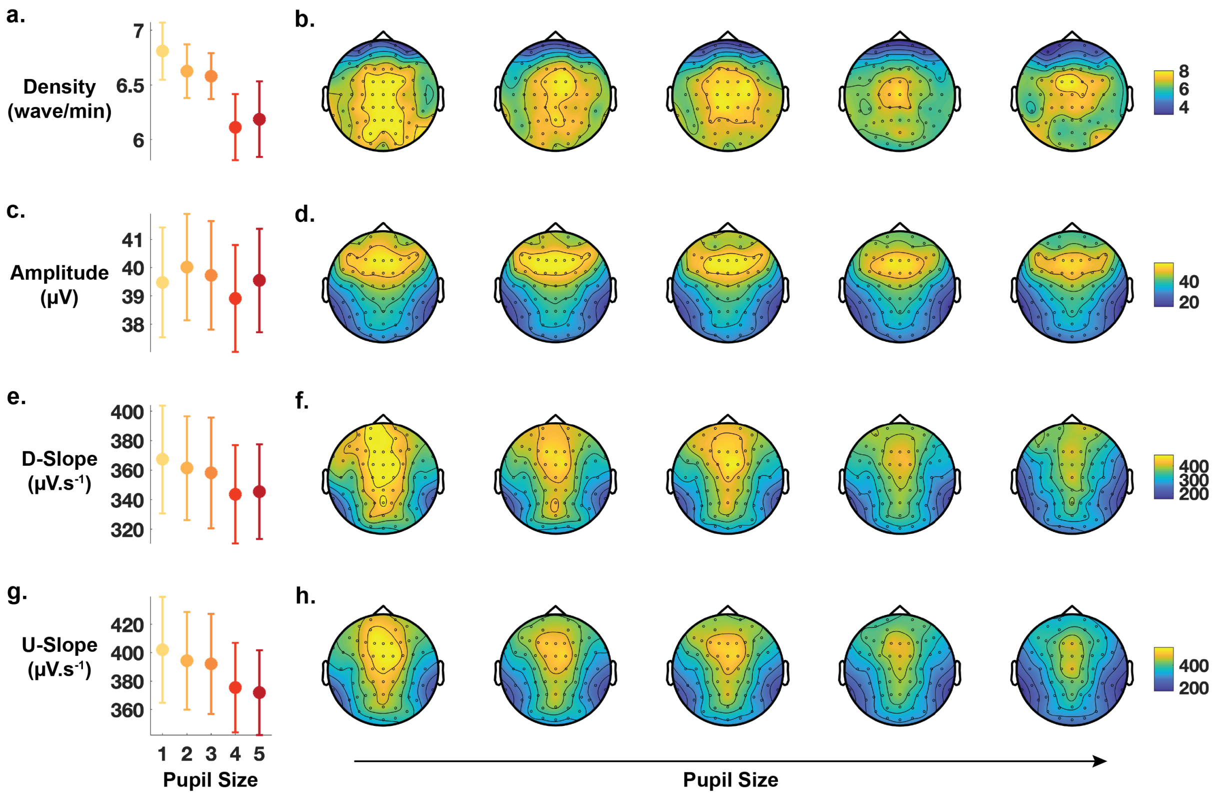

Supplementary Figure 2. A reduction in pupil size is associated with more, larger and steeper slow waves

Slow waves detected within 20s of probes' onset were separated according to participants' pupil size (binned, see Methods) computed on the same window. The density (a), amplitude (c), downward slope (D-Slope, e) and upward slope (U-Slope, f), averaged across participants and electrodes, are shown with error-bars showing the standard-error-of-the-mean across participants (N=25). Topographies of the average density (b), amplitude (d), downward slope (f) and upward slope (h) are also shown for the different pupil size bins.

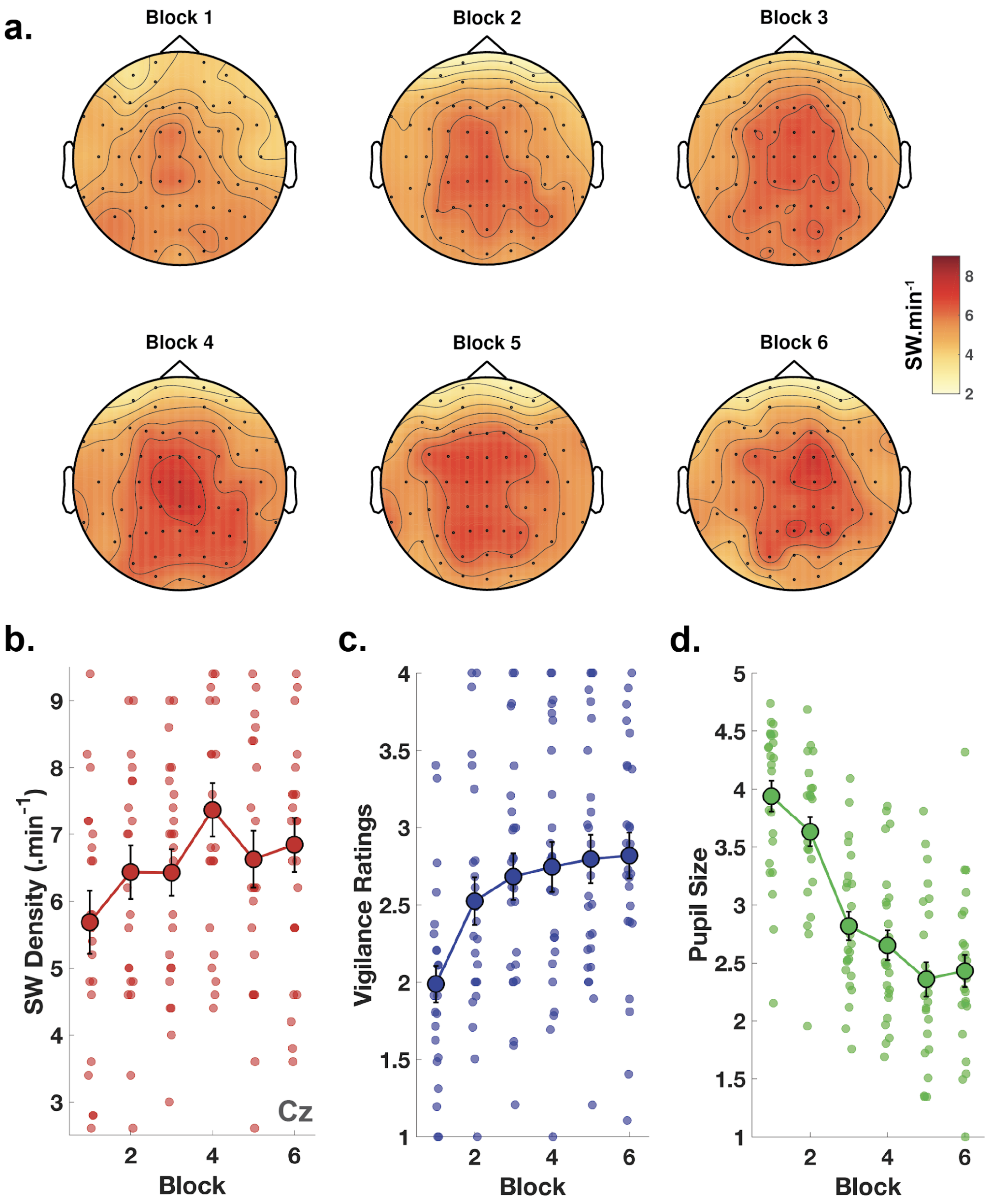

Supplementary Figure 3. Slow waves increase with time spent on task

(a) Topographies of the temporal density of slow waves (slow waves per minute, averaged across all 26 subjects). An increase in the number of slow waves, maximal over central electrodes, can be seen from the first to the last experimental block. (b-d) Slow wave density for electrode Cz (b), vigilance ratings (c) and pupil size (d) averaged within each block. Connected larger dots show the average across participants (error-bars: Standard-Error of the Mean (SEM); N=26, except for panel d: N=25). Individual semi-transparent circles show individual subjects.

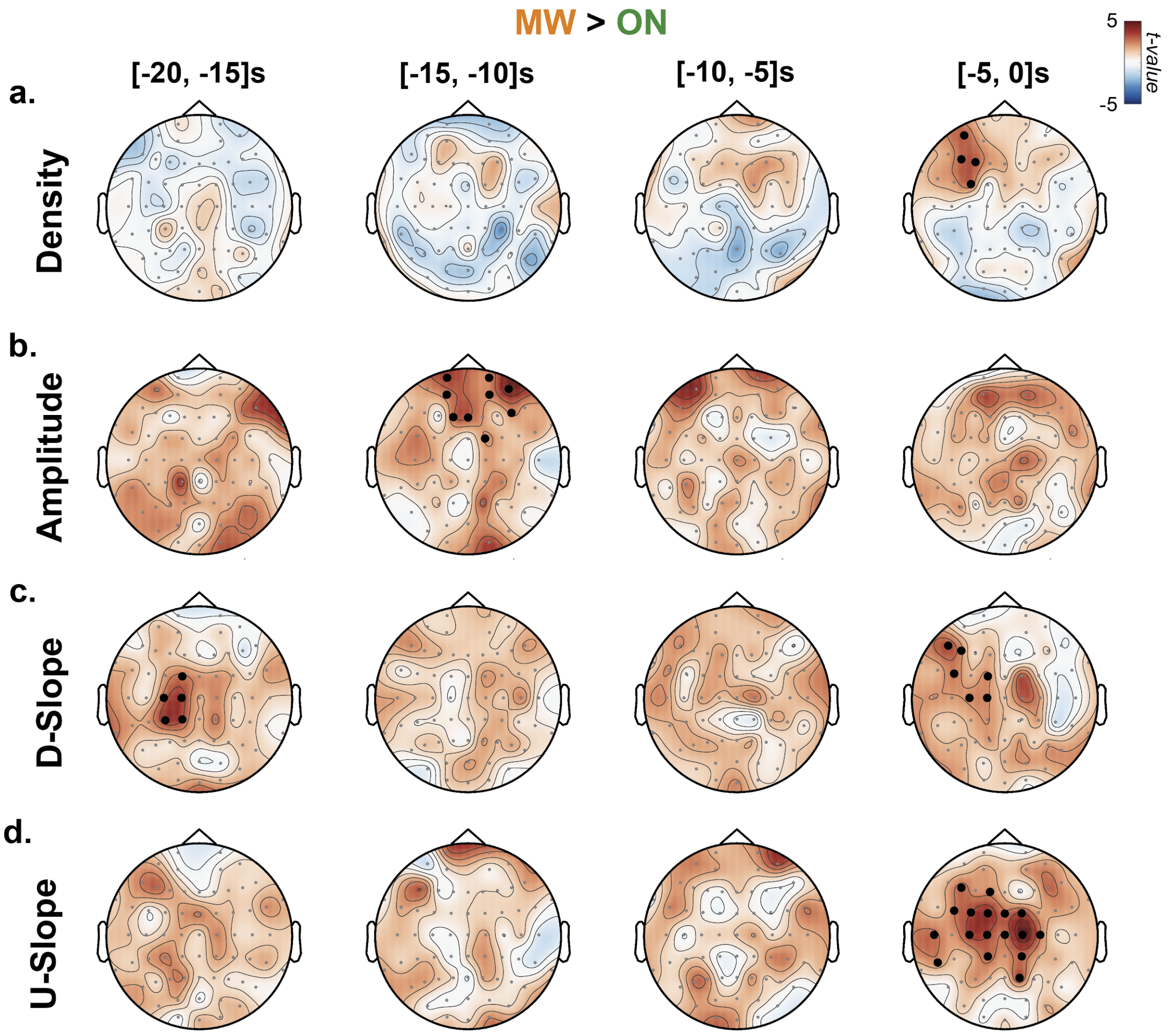

Supplementary Figure 4. Spatiotemporal dynamics of the effect of slow-waves properties on mental states (MW vs ON).

Mixed-Effects Models were used to quantify the impact of slow-wave properties (**a**: Density; **b**: Amplitude; **c**: Downward Slope (D-Slope); **d**: Upward Slope (U-Slope)) on mental states as in Figure 4. Slow waves were detected on 4 different windows: [-20, -15]s, [-15, -10]s, [-10, -5]s and [-5, 0]s before probe onsets. Slow-wave properties were extracted for each electrode and used to predict the MW (mind wandering) vs ON (task-focused) contrast. Topographies show the scalp distribution of the t-values associated with each slow-wave parameter and electrode. Black dots denote significant clusters of electrodes ($p_{cluster}$<0.05 corrected for 48 comparisons (Supplementary Figures 4-6) using a Bonferroni approach).

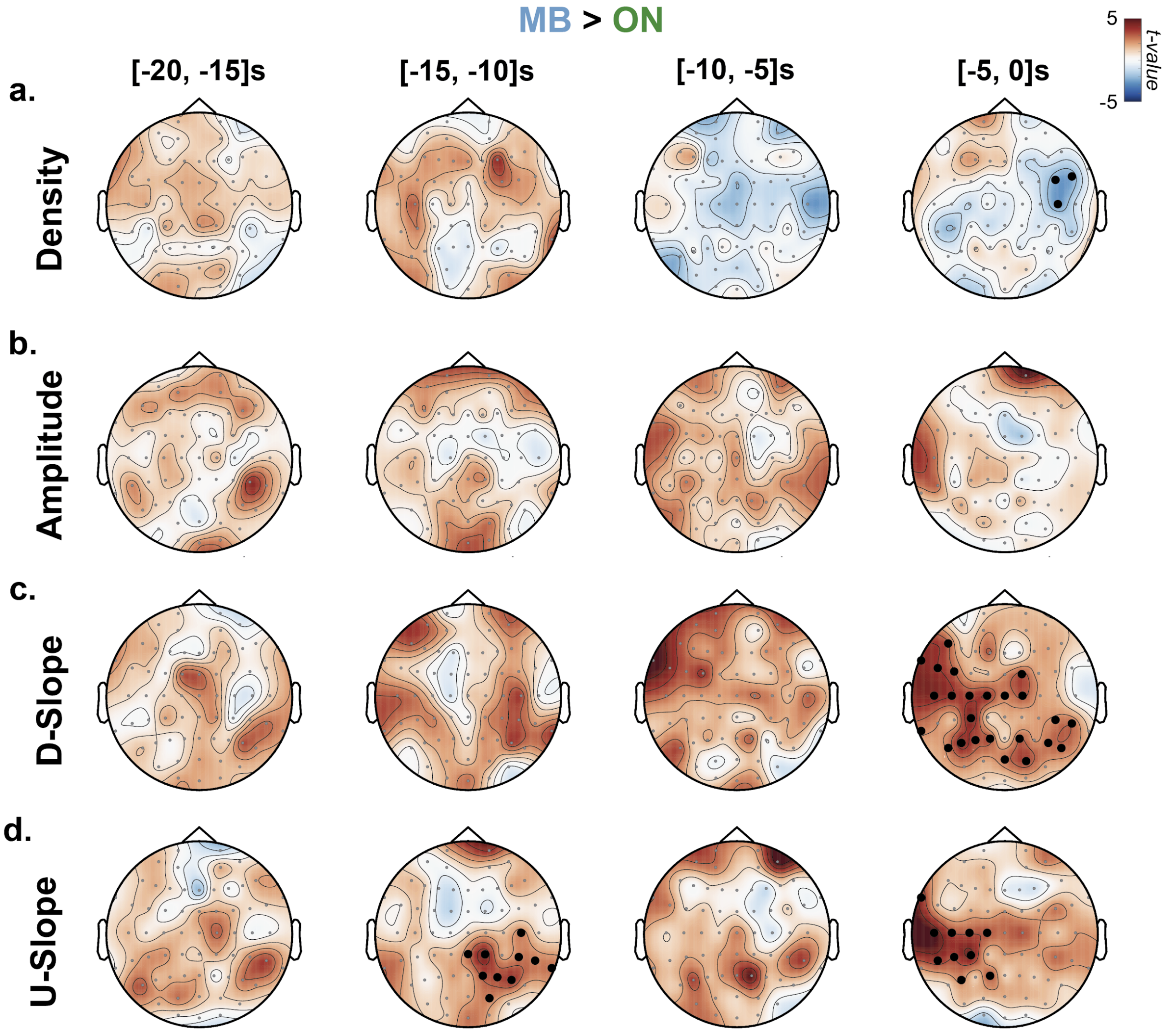

Supplementary Figure 5. Spatiotemporal dynamics of the effect of slow-waves properties on mental states (MB vs ON).

Same format as in Supplementary Figure 4. ON: task-focused; MB: mind blanking.

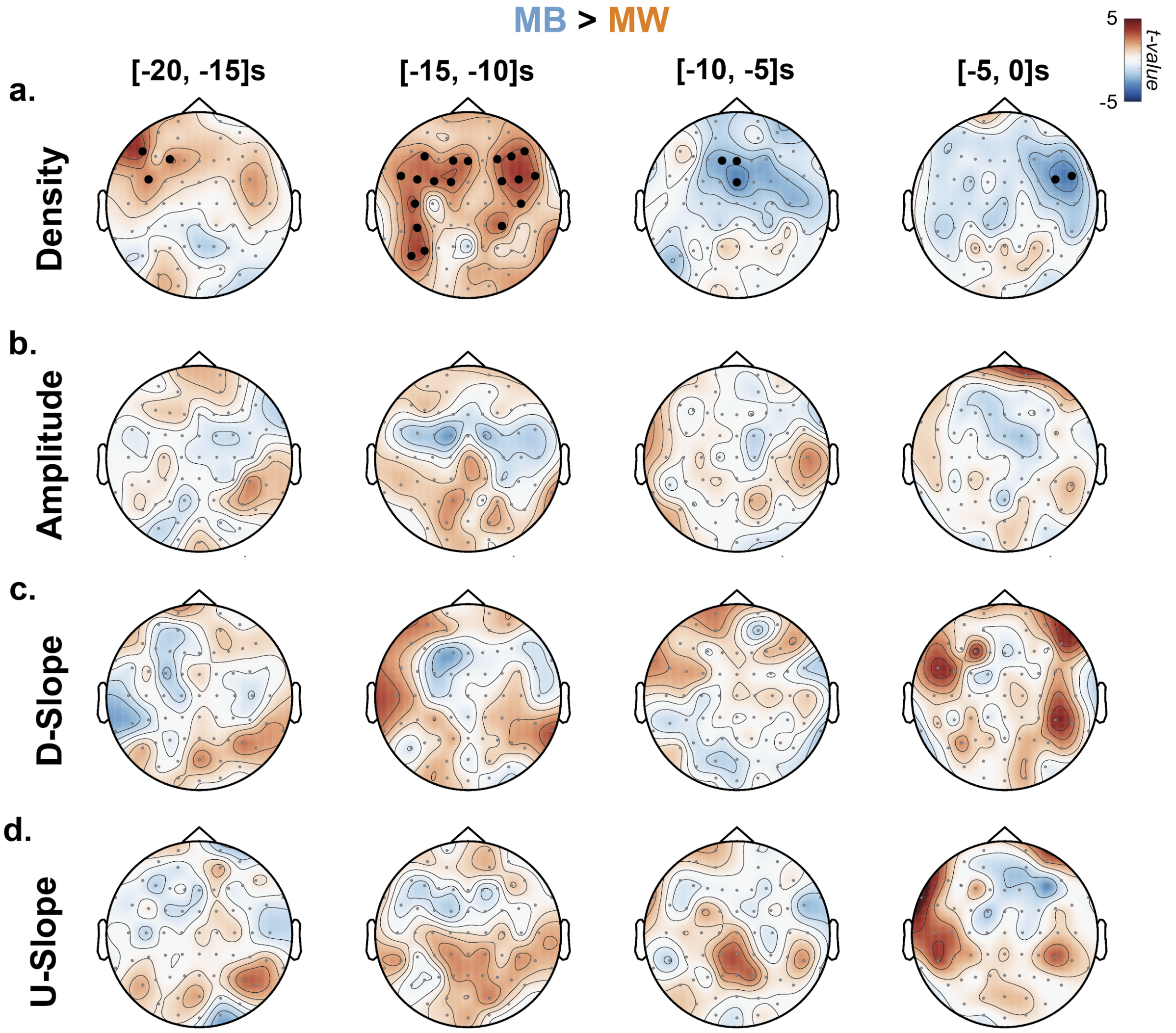

Supplementary Figure 6. Spatiotemporal dynamics of the effect of slow-waves properties on mental states (MB vs MW).

Same format as in Supplementary Figure 4. MW: mind wandering; MB: mind blanking.

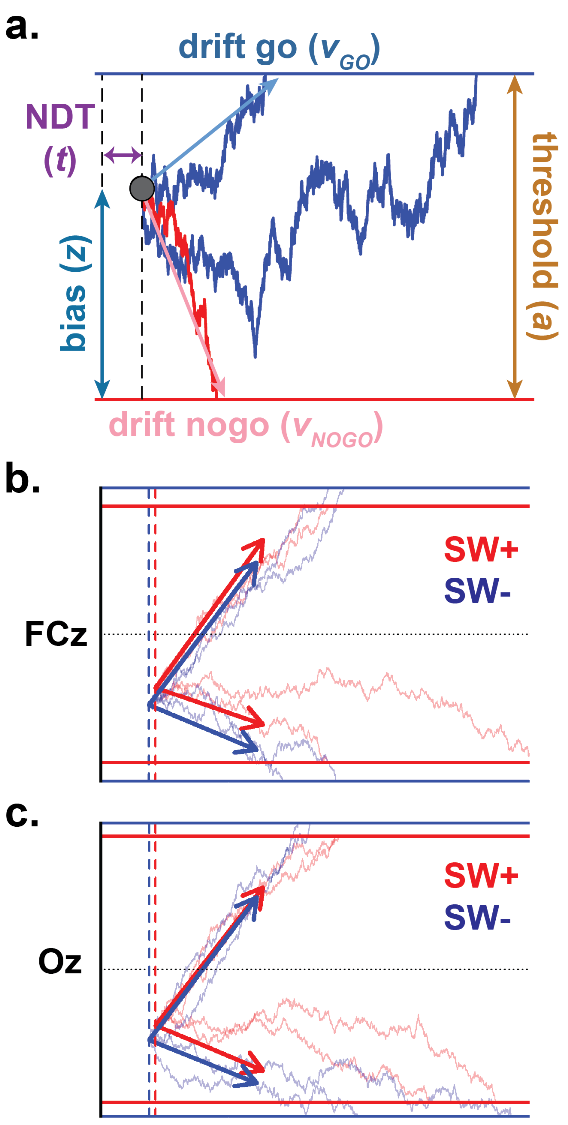

Supplementary Figure 7. Drift Diffusion Modelling

a: The Go/NoGo tasks were modelled according to the Drift Diffusion Model (DDM, see Methods and Supplementary Methods). The following parameters were estimated: threshold (*a*), non-decision time or NDT (*t*), bias (*z*), drift rate for Go trials (v_{Go}), drift rate for NoGo trials (v_{NoGo}). We then also computed the drift bias: abs(v_{Go})-abs(v_{NoGo}). The figure shows a graphical representation of these parameters. Note that here, drift rates for NoGo trials are negative. **b-c:** Graphical representation of decision processes using the parameters obtained by the DDM for trials with (SW+) or without (SW-) slow waves. The presence or absence of slow waves was determined for each electrode (FCz (b; frontal) and Oz (c; posterior)). Note the reduction in decision threshold, drift rates and bias associated with slow waves but the increase in NDT.

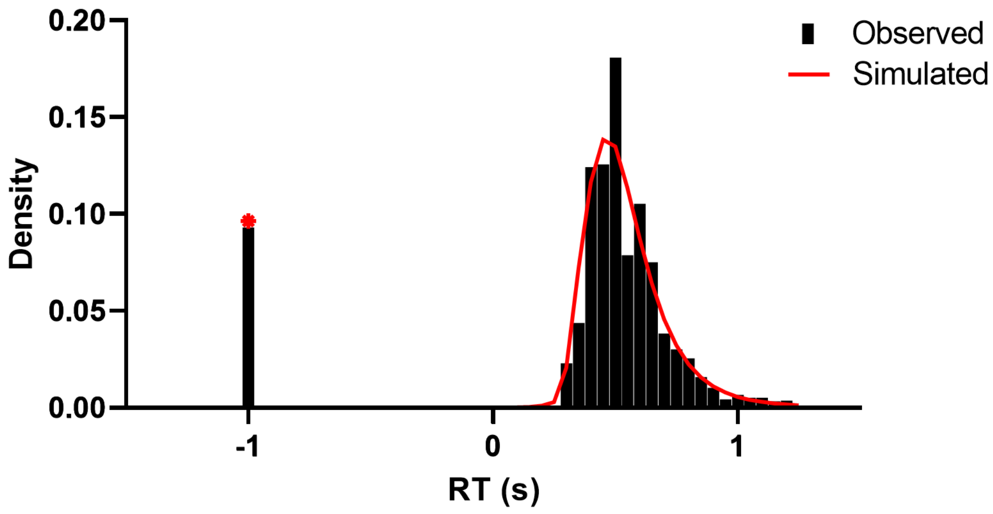

Supplementary Figure 8. HDDM Fit to Behavioural Data

Posterior predictive checks of Go/No-Go DDM fit to the behavioural data. Observed data for all participants (black bars, N=26) are plotted underneath model-predicted RT distributions and No-Go choice proportions (red lines). Positive distribution represents the normalised frequency of reaction times (RT) from Go responses. Negative bin at RT=-1 represents the proportion of No-Go responses.

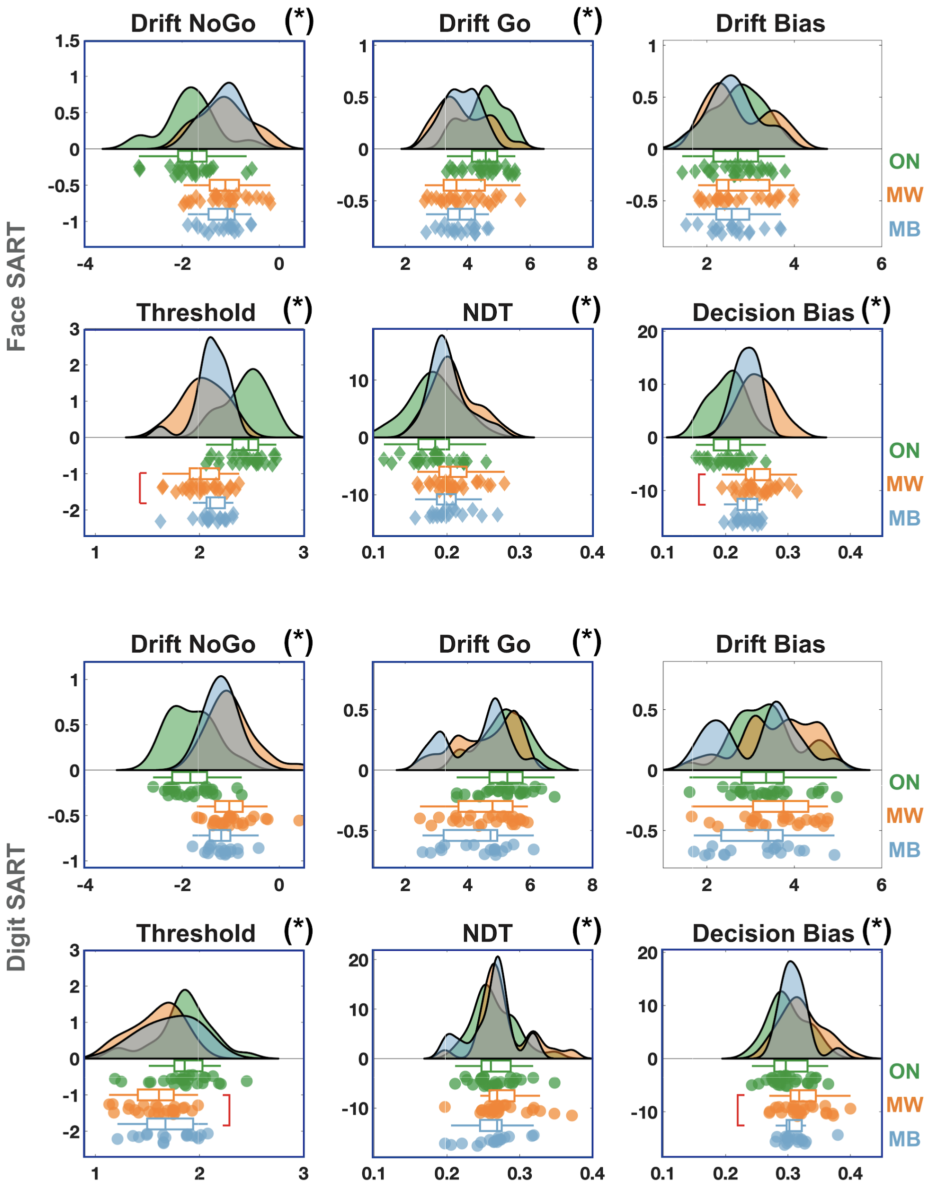

Supplementary Figure 9. Impact of mental states on HDDM parameters.

Hierarchical Drift Diffusion Modelling (HDDM, see Methods) was applied to the Reaction Times obtained in the Face (top) and Digit (bottom) SART (Sustained Attention to Response Task). The parameters were fitted for each task and mental state. Each panel shows the distribution of the estimated variable for individual participants (drift for NoGo and Go trials, drift bias, threshold, non-decision time (NDT) and decision bias; see Methods). For each plot, coloured areas show the smoothed distribution of individual data points (see Methods; N=26). Diamonds and circles show individual estimates for the Face and Digit SART respectively. Box plots show the 1st and 3rd quartiles (edges) as well as the median (middle bar). Blue boxes around individual plots and stars next to the titles indicate variables with significant state-effects (model comparison and two-tail Likelihood Ratio Test, see Methods; *: p<0.05, Bonferroni correction for 12 comparisons (p-values from left to right, top to bottom: p=1.2x10^{-5}; p=2.0x10^{-10}; p=0.45; p=2.5x10^{-13}; p=0.0096; p=7.0x10^{-12}; p=2.8x10^{-9}; p=2.3x10^{-6}; p=1; p=1.4x10^{-6}; p=0.48; p=0.02). Significant differences between MW and MB (for threshold and decision bias) are highlighted with red brackets. ON: task-focused; MW: mind wandering; MB: mind blanking.

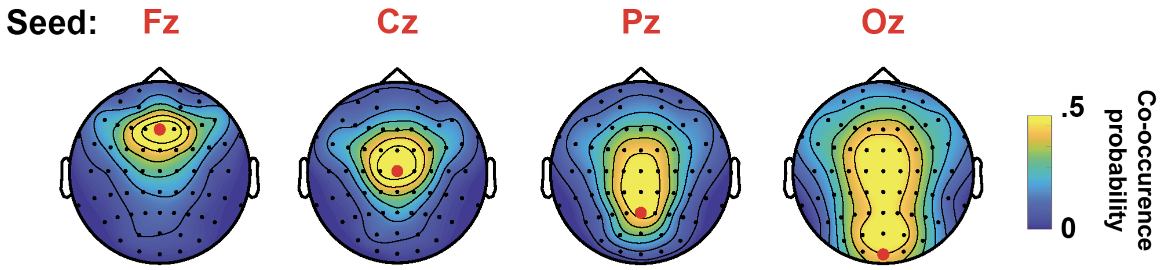

Supplementary Figure 10. Spatial expanse of slow waves

Four seeds electrodes were selected along the scalp midline from the front (Fz) to back (Oz). For each slow wave detected in these seed electrodes, we computed the probability that slow waves were also observed in the other electrodes. The average co-occurrence probability averaged across participants (N=26) is shown for each seed electrode. Note that slow waves detected over Fz tend to co-occur with other slow waves only in a limited number of neighbouring frontal electrodes whereas occipital slow waves (Oz) tend to co-occur with other slow waves in both frontal and posterior electrodes (more widespread).

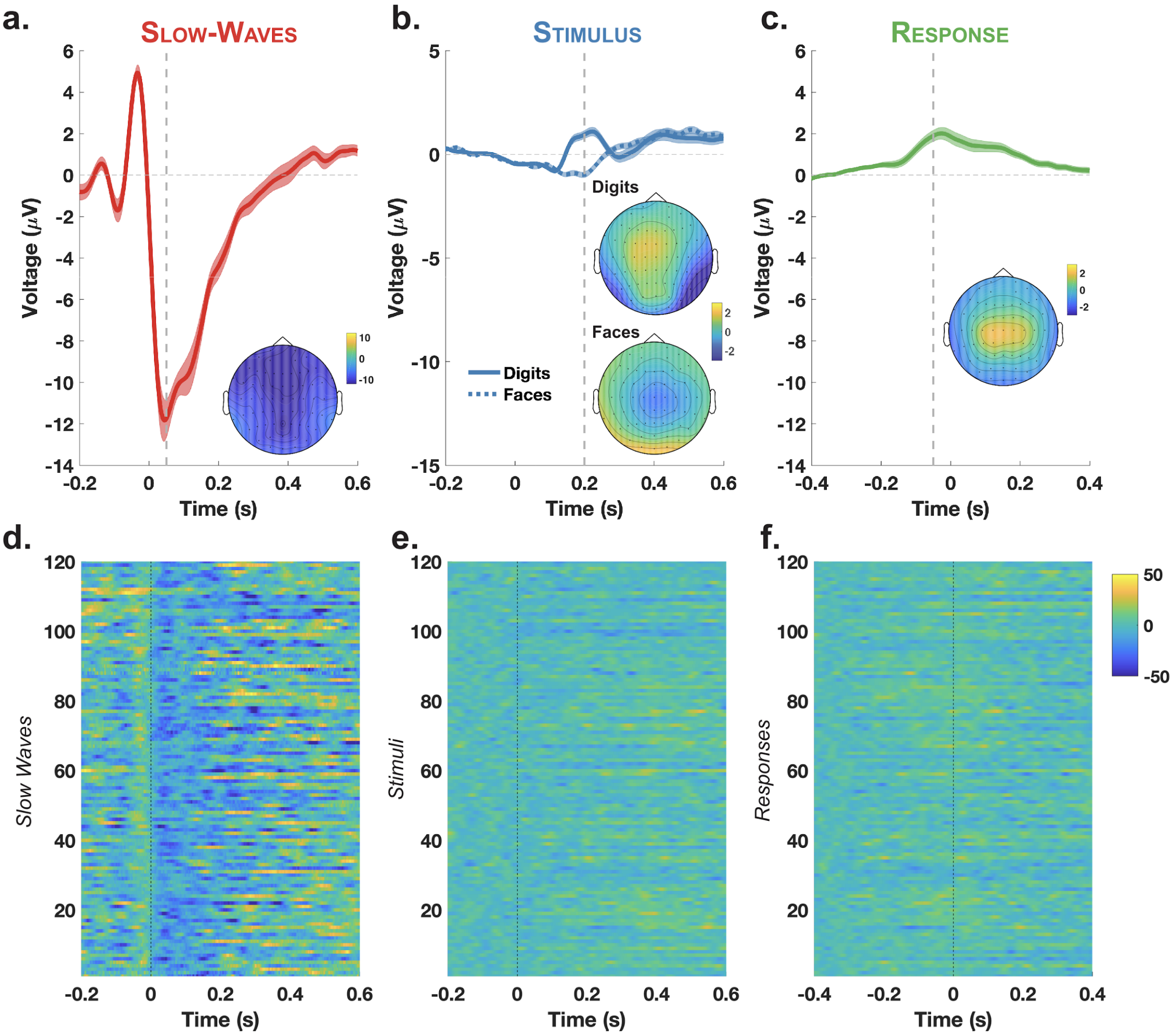

Supplementary Figure 11. Slow waves compared to stimulus and response-locked activity

Event-related potentials averaged over electrode Cz and across participants for slow waves (a), stimulus-locked activity (b) and response-locked activity (c). Slow waves' EEG time courses are aligned with the first negative crossing before the negative peak. Stimulus-locked responses are aligned on the onset of the digit (full line) or face (dotted line) stimuli. Response-locked responses are aligned to the onset of participants' responses. Shaded areas show the SEM (standard-error of the mean) across participants (N=26). Insets show the topography of the average voltage at the times shown by the vertical dotted lines (a: 0.05s; b: 0.2s; c: -0.05s), which approximates the absolute maximum of each ERP. (d-f) Voltage of the event-related potentials per trial for one participant. Each row corresponds to (d) an individual slow wave, (e) a stimulus presentation (face or digit), (f) a motor response. We limited the number of rows in (e) and (f) to match the number of detected slow waves (N=120) in (d).

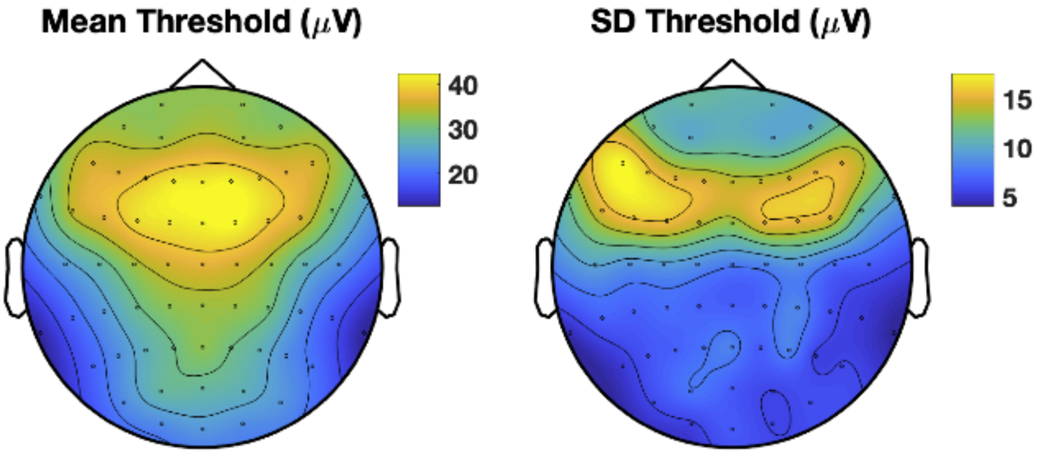

Supplementary Figure 12. Scalp topography of the average and standard-deviation of the slow-wave detection threshold

Only slow waves whose amplitude is within the top-10% of the distribution for each electrode and participant were analysed (N=26). The left topography shows the value, in voltage, of this threshold for each electrode, averaged across participants. The right topography shows the standard deviation (SD) of the threshold values across participants.

Supplementary References:

1. Mrazek, M. D., Phillips, D. T., Franklin, M. S., Broadway, J. M. & Schooler, J. W. Young and restless: validation of the Mind-Wandering Questionnaire (MWQ) reveals disruptive impact of mind-wandering for youth. *Frontiers in Psychology* **4**, (2013).
2. van Kempen, J. *et al.* Behavioural and neural signatures of perceptual decision-making are modulated by pupil-linked arousal. *eLife* **8**, (2019).
3. Iber, C., Ancoli-Israel, S., Chesson, A. & Quan, S. The AASM Manual for the Scoring of Sleep and Associated Events: Rules, Terminology and Technical Specifications. (2007).
4. Hung, C.-S. *et al.* Local experience-dependent changes in the wake EEG after prolonged wakefulness. *Sleep* **36**, 59–72 (2013).
5. Bernardi, G. *et al.* Neural and Behavioral Correlates of Extended Training during Sleep Deprivation in Humans: Evidence for Local, Task-Specific Effects. *Journal of Neuroscience* **35**, 4487–4500 (2015).
6. Quercia, A., Zappasodi, F., Committeri, G. & Ferrara, M. Local Use-Dependent Sleep in Wakefulness Links Performance Errors to Learning. *Frontiers in Human Neuroscience* **12**, (2018).
7. Legendre, G., Andrillon, T., Koroma, M. & Kouider, S. Sleepers track informative speech in a multitalker environment. *Nature Human Behaviour* **3**, 274–283 (2019).
8. Spiegelhalter, D. J., Best, N. G., Carlin, B. P. & van der Linde, A. Bayesian measures of model complexity and fit. *Journal of the Royal Statistical Society: Series B (Statistical Methodology)* **64**, 583–639 (2002).
9. Maris, E. & Oostenveld, R. Nonparametric statistical testing of EEG- and MEG-data. *J. Neurosci. Methods* **164**, 177–190 (2007).
