# Supplementary material for: Predicting lapses of attention with sleep-like slow waves
Source: Nat Commun. 2021 Jun 29;12:3657. doi: 10.1038/s41467-021-23890-7 (PMC8241869; doi:10.1038/s41467-021-23890-7)
Supplement: Supplementary file 3 — Reporting Summary [file 41467_2021_23890_MOESM3_ESM.pdf]

nature research

Corresponding author(s): Thomas Andrillon
Last updated by author(s): 3/26/2021

# Reporting Summary

Nature Research wishes to improve the reproducibility of the work that we publish. This form provides structure for consistency and transparency in reporting. For further information on Nature Research policies, see our Editorial Policies and the Editorial Policy Checklist.

## Statistics

For all statistical analyses, confirm that the following items are present in the figure legend, table legend, main text, or Methods section.

| n/a | Confirmed | |
|---|---|---|
| ☐ | ☒ | The exact sample size (*n*) for each experimental group/condition, given as a discrete number and unit of measurement |
| ☐ | ☒ | A statement on whether measurements were taken from distinct samples or whether the same sample was measured repeatedly |
| ☐ | ☒ | The statistical test(s) used AND whether they are one- or two-sided<br>*Only common tests should be described solely by name; describe more complex techniques in the Methods section.* |
| ☐ | ☒ | A description of all covariates tested |
| ☐ | ☒ | A description of any assumptions or corrections, such as tests of normality and adjustment for multiple comparisons |
| ☐ | ☒ | A full description of the statistical parameters including central tendency (e.g. means) or other basic estimates (e.g. regression coefficient) AND variation (e.g. standard deviation) or associated estimates of uncertainty (e.g. confidence intervals) |
| ☐ | ☒ | For null hypothesis testing, the test statistic (e.g. *F*, *t*, *r*) with confidence intervals, effect sizes, degrees of freedom and *P* value noted<br>*Give P values as exact values whenever suitable.* |
| ☐ | ☒ | For Bayesian analysis, information on the choice of priors and Markov chain Monte Carlo settings |
| ☒ | ☐ | For hierarchical and complex designs, identification of the appropriate level for tests and full reporting of outcomes |
| ☒ | ☐ | Estimates of effect sizes (e.g. Cohen's *d*, Pearson's *r*), indicating how they were calculated |

*Our web collection on statistics for biologists contains articles on many of the points above.*

## Software and code

Policy information about availability of computer code

| | |
|---|---|
| Data collection | Data collection was performed using the following softwares and codes:<br>- stimulus presentation and behavioral data collection: Psychtoolbox(3.0.14) running on Matlab (2018b) for Windows<br>- EEG data collection: BrainVision Recorder v1.21.0402 (BrainProducts)<br>- Eye-tracking data collection: Eyelink v5.09 for the EyeLink 1000 Plus (SR Research) |
| Data analysis | Data were analyzed in Matlab 2017b and Python2.8 using open-source toolboxes (SPM12, EEGlab (14.1.2b), FieldTrip (9bfe2f49f) and HDDM (0.6.0)) and custom codes (available on GitHub: https://github.com/andrillon/wanderIM). |

For manuscripts utilizing custom algorithms or software that are central to the research but not yet described in published literature, software must be made available to editors and reviewers. We strongly encourage code deposition in a community repository (e.g. GitHub). See the Nature Research guidelines for submitting code & software for further information.

## Data

Policy information about availability of data

All manuscripts must include a data availability statement. This statement should provide the following information, where applicable:
- Accession codes, unique identifiers, or web links for publicly available datasets
- A list of figures that have associated raw data
- A description of any restrictions on data availability

All raw data are publicly available on the website of the Open Science Framework (https://osf.io/ey3ca/?view_only=680c39e7065649c3b783a4efec0a1a94).

# Field-specific reporting

Please select the one below that is the best fit for your research. If you are not sure, read the appropriate sections before making your selection.

☐ Life sciences ☒ Behavioural & social sciences ☐ Ecological, evolutionary & environmental sciences

For a reference copy of the document with all sections, see nature.com/documents/nr-reporting-summary-flat.pdf

# Behavioural & social sciences study design

All studies must disclose on these points even when the disclosure is negative.

| | |
|---|---|
| Study description | The study is a within-subject design exploring the impact of physiological indexes (quantitative variables) on behavioral performance (quantitative variables) and subjective reports (qualitative variables). |
| Research sample | Participants were healthy adults (32 tested, 26 included in the analyses). The remaining 26 participants (age: 29.8 ± 4.1 years, mean ± standard-deviation; 10 females) can be considered representative of the Australian, urban, educated population. The study sample was chosen precisely to be representative of healthy adults in an industrialized country. |
| Sampling strategy | There was no a priori strategy to select the sample size as there was no relevant prior study to perform power computations. The size of the sample was chosen within the upper bound of similar EEG studies (Braboszcz et al. Neuroimage 2011, van Son et al. 2019, n=26,Yi Jin et al. 2019). |
| Data collection | High-density scalp electroencephalography (EEG) was recorded using an EasyCap (63 active electrodes) connected to a BrainAmp system (Brain Products GmbH). Eye-movements and pupil size on one eye were recorded with an EyeLink 1000 system (SR Research) with a sampling frequency of 1000Hz. Participants' behaviur was recorded using a keyboard and the Psychtoolbox running on Matlab. Only the researcher performing data collection was present and in a separate room during testing. The researcher was not blind to the hypothesis. |
| Timing | Data was collected in two time periods (June 2018 and December 2018/Januaray 2019). |
| Data exclusions | Six individuals were not included in our analyses because of technical issues during recordings or an abnormal quality of physiological recordings assessed through a post-hoc visual inspection of the data. |
| Non-participation | One participant (not included in the total) agreed to participate to the experiment but did attend their apointment for the recording session. They did not provide a justification. |
| Randomization | All participants followed the same procedure. The order of test blocks was randomised for each participants (function randperm.m in Matlab). The temporal interval between trials and probes was also randomly selected using the function rand.m in Matlab (see Methods). |

# Reporting for specific materials, systems and methods

We require information from authors about some types of materials, experimental systems and methods used in many studies. Here, indicate whether each material, system or method listed is relevant to your study. If you are not sure if a list item applies to your research, read the appropriate section before selecting a response.

## Materials & experimental systems

| n/a | Involved in the study | |
|---|---|---|
| ☒ | ☐ | Antibodies |
| ☒ | ☐ | Eukaryotic cell lines |
| ☒ | ☐ | Palaeontology and archaeology |
| ☒ | ☐ | Animals and other organisms |
| ☐ | ☒ | Human research participants |
| ☒ | ☐ | Clinical data |
| ☒ | ☐ | Dual use research of concern |

## Methods

| n/a | Involved in the study | |
|---|---|---|
| ☒ | ☐ | ChIP-seq |
| ☒ | ☐ | Flow cytometry |
| ☒ | ☐ | MRI-based neuroimaging |

## Human research participants

Policy information about studies involving human research participants

| | |
|---|---|
| Population characteristics | See above. |
| Recruitment | Participants were recruited within a professional network and through the researcher in charge of data collection (TM). This specific recruitment procedure was not related to our hypotheses. Participants were not informed about the aims of this study and our hypothesis. They were just informed that we aimed at studying attention. Self-selection bias could include curiosity about cognition and the brain. As no information was provided about the study's aims, it is unlikely that such biases could have impacted our results. |
| Ethics oversight | The protocol was approved by the Monash University Human Research Ethics Committee (Project Number: 10994). Participants provided written informed consent. |

Note that full information on the approval of the study protocol must also be provided in the manuscript.
